# A Sensitivity-Enhanced Refractive Index Sensor Using a Single-Mode Thin-Core Fiber Incorporating an Abrupt Taper

**DOI:** 10.3390/s120404697

**Published:** 2012-04-11

**Authors:** Jie Shi, Shilin Xiao, Lilin Yi, Meihua Bi

**Affiliations:** State Key Lab of Advanced Optical Communication Systems and Networks, Department of Electronics Engineering, Shanghai Jiao Tong University, No. 800, Dongchuan Road, Minhang District, Shanghai 200240, China; E-Mails: slxiao@sjtu.edu.cn (S.X.); lilinyi@sjtu.edu.cn (L.Y.); huahuabi2006@sjtu.edu.cn (M.B.)

**Keywords:** refractive index sensor, inter-model interferometer, fiber taper

## Abstract

A sensitivity-enhanced fiber-optic refractive index (RI) sensor based on a tapered single-mode thin-core diameter fiber is proposed and experimentally demonstrated. The sensor head is formed by splicing a section of tapered thin-core diameter fiber (TCF) between two sections of single-mode fibers (SMFs). The cladding modes are excited at the first SMF-TCF interface, and then interfere with the core mode at the second interface, thus forming an inter-modal interferometer (IMI). An abrupt taper (tens of micrometers long) made by the electric-arc-heating method is utilized, and plays an important role in improving sensing sensitivity. The whole manufacture process only involves fiber splicing and tapering, and all the fabrication process can be achieved by a commercial fiber fusion splicer. Using glycerol and water mixture solution as an example, the experimental results show that the refractive index sensitivity is measured to be 0.591 nm for 1% change of surrounding RI. The proposed sensor structure features simple structure, low cost, easy fabrication, and high sensitivity.

## 1. Introduction

Fiber-optic refractive index (RI) sensors are of considerable interest due to their small size, high sensitivity, immunity to electromagnetic interferences, and flexibility of directly embedding into the system structure. A number of optical fiber RI sensors have been developed, such as fiber Bragg gratings (FBGs) [1–5], long-period gratings (LPGs) [6,7], and LPG pairs [8]. Sensor configurations fabricated by femtosecond laser pulses have also been proposed for use in RI sensing [9]. FBGs always use removal of the fiber cladding to increase the sensing sensitivity. The cladding mode of LPG is sensitive to the RI of the surrounding medium, thus allowing it to be employed as a RI sensor. However, the broad transmission resonance (typically tens of nanometers) limits the measurement accuracy. In addition, grating-based sensors (FBG, LPG) require precise and expensive phase masks and photolithographic procedures. Recently, in-line fiber inter-modal interferometers (IMIs) have been studied for RI sensing. Among them, core mismatch [10,11], fiber tapers [12], and photonic crystal fibers [13,14] have been used to form interferometers in RI sensing applications. IMIs have several favorable features, including simple fabrication, low cost, and high sensing sensitivity.

In this paper, we present an improved in-line IMI for highly sensitive RI sensing, which is formed by simply splicing a tapered thin-core diameter fiber (TCF) between two sections of single-mode fibers (SMFs). In the proposed sensor structure, new cladding modes are excited by the abrupt taper, and the orders of the new cladding modes are higher than that of the dominantly cladding mode. For the IMI-based RI sensor, the higher order cladding modes generally exhibit higher sensitivities. That is because high order cladding modes have larger mode field area, and are more easily affected by the surrounding RI. Therefore, the sensing sensitivity can be improved. The fiber taper region is very short (less than 1 mm), yet it can significantly enhance the sensitivity of the sensor. Using glycerol and water mixture solutions as examples, the maximum wavelength shift of 0.591 nm is measured for an RI change of 0.01, which is about two times higher than that of TCF with no taper. The simple sensing structure offers the advantages of low cost, simple fabrication, and high sensitivity, and may thus find potential applications in chemical or biological sensors.

## 2. Operation Principle

**Figure 1.**
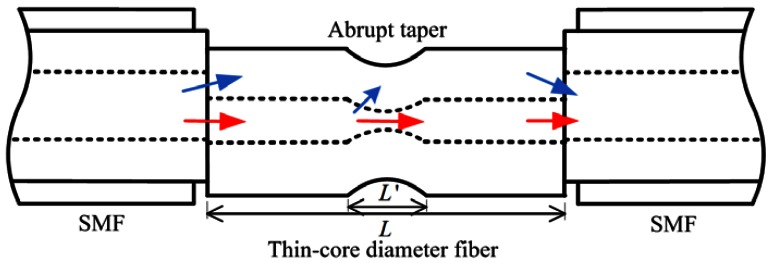
Schematic diagram of the proposed sensor; *L and L′* are the length of the TCF and the taper length, respectively.

The structure of the proposed RI sensor is shown in Figure 1. A section of uncoated commercial TCF is spliced between two sections of SMFs. At the SMF-TCF interface, part of the light can be coupled to the cladding of the TCF as cladding modes due to mode mismatch. After propagating through the TCF, the excited cladding modes will be re-coupled to the core of the SMF at the second spliced point. The cladding modes will interfere with the core mode due to the phase difference, which can be described as:

(1) $$\Phi^{j}=\frac{2\pi (n_{\text{core}}^{\text{eff}}-n_{cl,j}^{\text{eff}})L}{\lambda }=\frac{2\pi \Delta n_{j}^{\text{eff}}L}{\lambda }$$

where ${\text{n}}_{\text{core}}^{\text{eff}}$ and ${\text{n}}_{\text{cl},\text{j}}^{\text{eff}}$ are the effective refractive index of the core and the *j*th cladding modes, respectively; $\Delta n_{j}^{\text{eff}}$ is the difference between effective RIof the core mode and the *j*th cladding mode; *L* is the interference length and λ is the light wavelength in vacuum. The intensity of the interference signal *I* is:

(2) $$I=I_{1}+I_{2}+2\sqrt{I_{1}I_{2}}\cos(\Phi^{j})$$

where *I*_1_ and *I*_2_ are the intensities of the lights propagating along the fiber core and cladding, respectively. The typical extinction ratio for the IMI is generally small without adopting an offset between the SMF and the TCF. In order to obtain a high interference depth, we slightly offset a splice between the SMF and the TCF.

The intensity of the interference signal reaches its transmission dips when Φ becomes an odd times of π. Then, Equation (1) can be written as:

(3) $$\lambda_{D}=\frac{2\Delta n_{j}^{\text{eff}}L}{2n+1}$$

where λ_D_ is the wavelength of transmission dip, *n* is an integer. If the RI of the surrounding medium increases, the effective RIof the cladding mode increases, while that of the core mode stays the same value. When $\Delta n_{\text{j}}^{\text{eff}}$ j decreases by $\delta n_{\text{cl},\text{j}}^{\text{eff}}$, λ_D_ will shift to the shorter wavelength by *δλ*_D_:

(4) $$\delta \lambda_{D}\approx 2L\delta n_{cl,j}^{\text{eff}}$$

Based on Equation (4), it is possible to measure the RI of the surrounding medium by monitoring the resonance wavelength change of *δλ*_D_. Equation (4) also indicates that the sensitivity of the interferometer is dependent on both the length *L* and the effective RI change of the cladding mode. Since too long a sensor (large *L*) would make it difficult to make a compact sensor, we use the method of exciting the higher order cladding modes to improve the sensing performance. For a specific RI change of the surrounding medium, the effective RI change of a higher order cladding mode is larger than that of a lower order mode, which means that the sensitivity of the RI sensor can be improved by exciting the higher order cladding mode [15]. Fiber taper structures have been extensively studied in SMFs and photonic crystal fibers [16,17]. For such applications more gradual tapers with smaller taper angles are preferred, because they are focused on reducing the insertion loss, while for abrupt tapers or larger taper angles, light in the core will be partially coupled into the cladding of the fiber as cladding modes. SMF typically supports only the fundamental mode (LP_01_) in the core, while the other higher order linear polarization modes mainly propagate as cladding modes. For the gradual fiber taper with small taper angle, the relative change in the taper radius is very small, so the main portion of the power remains in the LP_01_ mode and does not couple with other higher order cladding modes as it propagates along the taper. But for an abrupt taper, the coupling occurs between the LP_01_ mode and the higher order cladding modes. In our experiment, a short but abrupt taper is made in the TCF to excite higher order cladding modes. Compared with the total length of the interferometer, the taper zone is small, yet is sufficient to enhance the sensing sensitivity.

## 3. Experiments and Discussion

### 3.1. Sensor Fabrication

**Figure 2.**
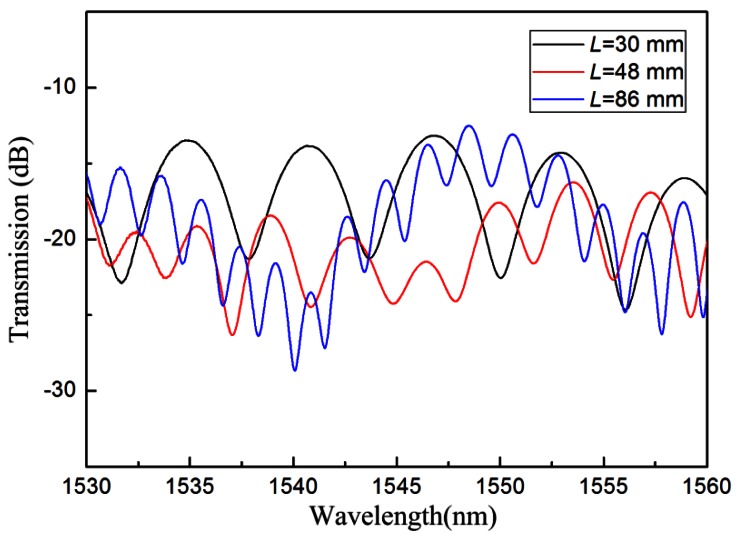
Transmission spectra of the IMIs with different TCF lengths.

The TCF used in our experiment is a commercial fiber (Yangtze Ltd., Wuhan, China). It has core/cladding diameters of 4.8/125 μm, and the cut-off wavelength is 1,260 nm. An Ericsson fusion splicer (modal FSU 975), with a built-in core-offset attenuator program, was used to produce the lateral offset between the SMF and the TCF. A C-band light source was used as the broad band source, and the output spectrum was monitored by an optical spectrum analyzer (OSA, Yokogawa, Japan) with wavelength resolution of 15 pm. The transmission spectra of the IMIs with different TCF lengths were measured and are shown in Figure 2. It can be seen that the free spectral range decreases with the increasing interference length. In addition, the interference spectra are a little inhomogeneous. This is because that more than one cladding modes are available in the interference pattern.

**Figure 3.**
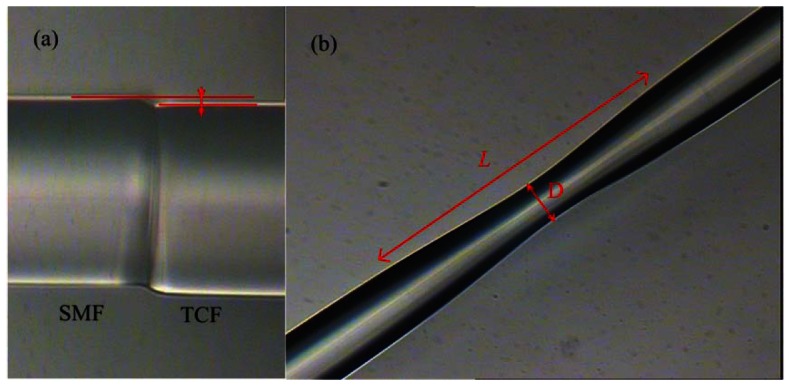
(**a**) Photograph of the lateral core offset between the SMF and TCF, the offset is ∼5 μm; (**b**) The taper range in the TCF, *L* = 697 μm, *D* = 91 μm.

The short taper in the TCF was made by using electric-arc-heating method, which can also be achieved by the same fiber fusion splicer with the built-in taper program. Figure 3(a) shows a photograph of the core offset between the SMF and the TCF, and the offset is approximately 5 μm. Figure 3(b) shows the typical photograph of taper region. The taper length and waist diameter are 697 μm and 91 μm, respectively.

### 3.2. RI Measurement and Discussion

**Figure 4.**
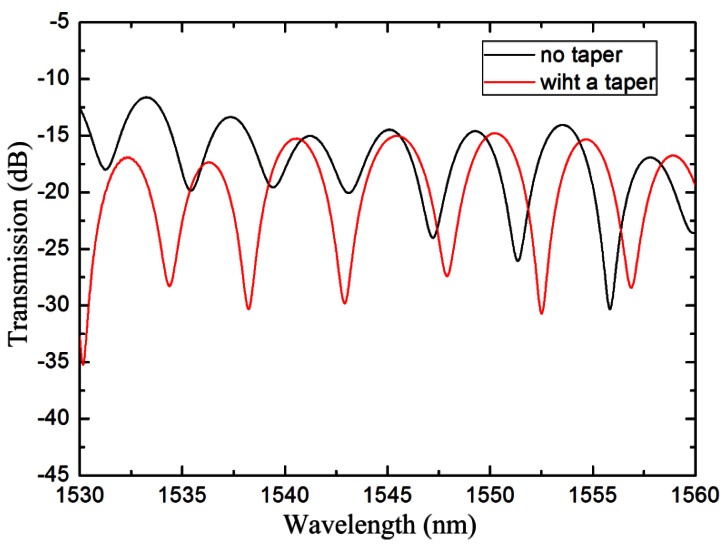
Measured transmission spectra of the IMI with a taper (red line) and with no taper (black line).

An IMI was fabricated with 38 mm length TCF, and was used as the sensor head. An abrupt taper, located in the center of the TCF, was made to improve the sensing sensitivity. The taper length and waist diameter were 697 μm and 91 μm, respectively. The transmission spectra of the IMI before and after the TCF was tapered are shown in Figure 4. After being tapered, the transmission dips change due to the variation of the waveguide structure. Moreover, the extinction ratio of the transmission spectrum enlarges. As shown in Equation (2), the interference visibility shows high value when the intensity of *I*_1_ and *I*_2_ are comparable. In particular, the *I*_min_ reaches to zero when *I*_1_ equals to *I*_2_. After tapering in the TCF, part of the core mode is coupled into cladding as cladding modes. The intensity of the cladding modes thus becomes more comparable with that of the core mode, therefore, the resonance intensity enlarges after the TCF being tapered.

**Figure 5.**
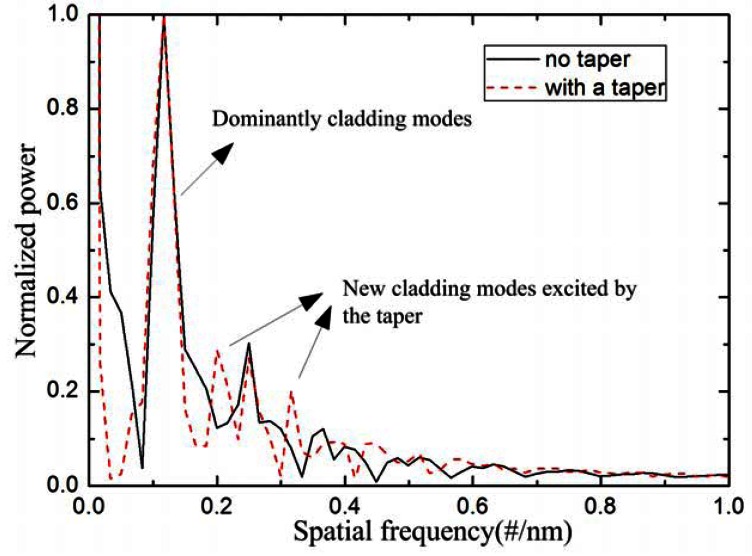
Spatial frequency spectra of the IMI with a taper (solid line) and with no taper (dashed line).

The interference patterns in Figure 4 are Fourier transformed to obtain the spatial frequency, as shown in Figure 5. It can be found that there is only one dominantly cladding mode, along with other weakly cladding modes. Take the solid line as an example, the dominantly cladding mode is excited at the spatial frequency of 0.116 #/nm. Meanwhile, other weakly cladding modes are also excited with the spatial frequencies of 0.249 #/nm, 0.366 #/nm, *etc*. After tapering the TCF, two new cladding modes (spatial frequency at 0.199 #/nm & 0.316 #/nm) are excited, whose orders are higher than that of the dominantly cladding mode because their spatial frequencies are larger than the dominantly cladding mode [18]. The new excited cladding modes will slightly change the main interference pattern. Wu *et al.* [15] simulated the effective RI change of the cladding mode for the same RI change of the surrounding medium. The effective RI change of the LP_0,10_ is 25 times higher than that of LP_0,3_, which means that the higher order cladding modes exhibit higher RI sensitivity. Due to the higher order cladding modes being excited, the RI sensitivity can be improved by adopting the abrupt taper in the TCF.

**Figure 6.**
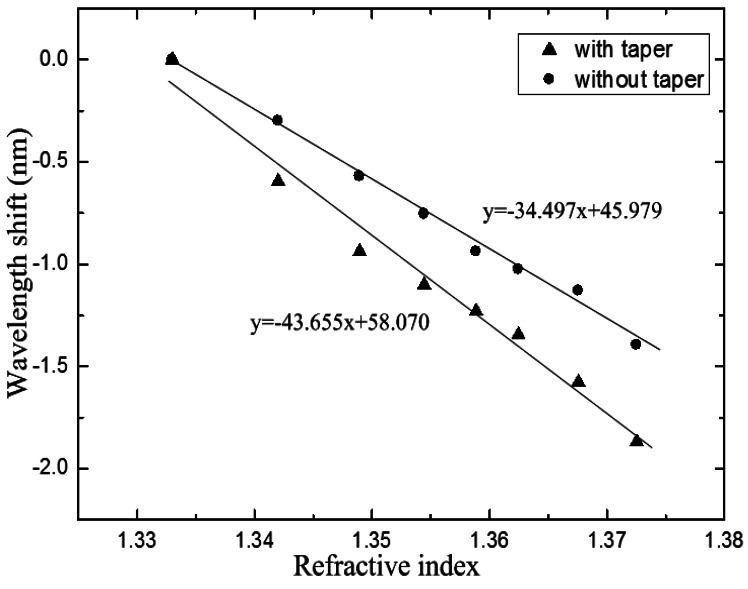
Wavelength shifts of the sensor with no taper (black dots) and with a taper (black triangles).

The IMI with the TCF length of 38 mm was employed to evaluate its RI sensitivity. We used solutions of glycerol and water mixture as samples, whose RI ranged from 1.333 to 1.3725. The refractive indices of the samples were measured using an Abbe refractometer with the wavelength of 589 nm, and the accuracy of the refractometer was 0.001. The sensor head was mounted on a glass plate with glue to keep it straight, and it was immersed into the mixed solutions. The entire measurement was carried out under a constant temperature to avoid the impact of temperature. The spectral responses to different RI values were recorded by the OSA. The IMI was carefully cleaned with alcohol and water after each RI measurement. Then, the sensor head was tapered in the center of the TCF. In order to make a comparison, the RI experiment was repeated by using the same sensor head after the TCF was tapered. The operating procedure was as same as that of the sensor head with no taper. The maximum wavelength shifts of the transmission dips with the variation of the RI are plotted in Figure 6. The correlation coefficient squares reached 0.994 and 0.978, respectively. The result shows that the RI sensitivity can be significantly improved by tapering the TCF. And the wavelength shift is enhanced from −0.345 nm to −0.437 nm for 0.01 change of RI.

**Figure 7.**
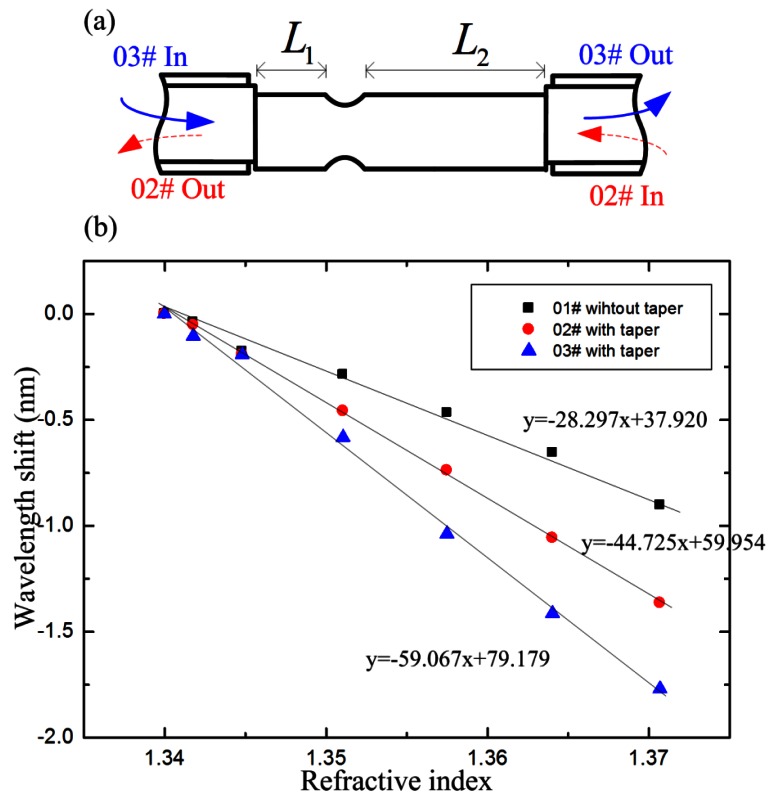
(**a**) Schematic diagram of the sensor head whose taper is not located in the center of the TCF; (**b**) Measured wavelength shifts with respect to RI change.

To characterize the impact of the taper position on RI sensitivity, we have fabricated another IMI, and the taper area was not in the center of the TCF, as shown in Figure 7(a). The taper has the length of ∼750 μm and the twist diameter is ∼80 μm; *L*_1_ and *L*_2_ are 13.5 mm and 17.7 mm, respectively. We did three groups of RI experiments with the same sensor head. Firstly, the IMI was used as a RI sensor before being tapered (01#). Secondly, the light was led in from the right side of the sensor after the TCF was tapered (02#). Thirdly, the light was led in from the left side of the sensor (03#). The spectral responses are plotted in Figure 7(b). The sensitivities of the three groups of RI experiments are −0.283 nm, −0.447 nm and −0.591 nm for 1% change of RI, respectively. Correlation coefficient squares of 0.991, 0.999 and 0.996 were obtained. Figure 7(b) indicates that the sensing sensitivity can be significantly improved by tapering the TCF. The sensing sensitivity is also affected by the taper position in the TCF. The wavelength shifts of 02# and 03# can be written as $\delta \lambda_{2}=2L_{1}\delta_{cl}^{\text{eff}}$, and $\delta \lambda_{3}=2L_{3}\delta_{cl}^{\text{eff}}$. Due to the symmetrical structure of the abrupt taper, the new excited cladding modes for 02# are suppose to show same sensitivity to the surround medium. Thus, the $\delta_{cl}^{\text{eff}}$ of 02# equals to that of 03#. *L*_2_>*L*_1_, so *δλ*_2_>*δλ*_3_. As a result, the sensing sensitivity of 02# is higher than that of 03#.

The above experimental results show that the sensitivity can be distinctly enhanced by slightly tapering the TCF. It is worth noting that the RI sensitivity of the proposed sensor could be enhanced by using a longer taper with a smaller waist diameter. However, too small a waist diameter of the taper would reduce the robustness of the sensor device. The ratio of the wavelength shift with respect to the wavelength spacing of the interference spectrum is used to evaluate the sensing performance. The value of the ratio for our proposed sensor (λ_shift_: 0.591 nm, wavelength spacing: 5 nm, ratio: 0.118) is larger than that of the LPG pair sensor (λ_shift_: 0.259 nm, wavelength spacing: 4 nm, ratio: 0.065) [8]. In addition, the proposed sensor also features the advantages of relatively low cost and easy fabrication.

## 4. Conclusions

We have demonstrated a cost-effective in-line IMI-typed RI sensor made by sandwiching a section of tapered thin core diameter fiber between SMFs. Although the taper is short, it can significantly improve the sensitivity of the proposed sensor. In addition, the whole manufacturing process (including the splicing and tapering processes) can be accomplished with a commercial fiber fusion splicer. Since the proposed RI sensor has the advantages of easy fabrication, high sensitivity, and low cost, it may find potential applications in the chemical and biological sensing fields.
